# PROPRIOCEPTIVE NEUROMUSCULAR FACILITATION THERAPY VERSUS MANUAL THERAPY IN PATIENTS WITH NECK PAIN: A RANDOMIZED CONTROLLED TRIAL

**DOI:** 10.2340/jrm.v56.40002

**Published:** 2024-09-05

**Authors:** Tomasz MAICKI, Rafał TRĄBKA, Magdalena WILK-FRAŃCZUK, Weronika KRZEPKOWSKA

**Affiliations:** 1 Rehabilitation Clinic, Institute of Physiotherapy, Faculty of Health Sciences, Jagiellonian University Medical College, Kraków, Poland; 2 Department of International Cooperation, Polish Chamber of Physiotherapists

**Keywords:** chronic pain, neck pain, physiotherapy, rehabilitation

## Abstract

**Objective:**

To compare the effects of proprioceptive neuromuscular facilitation therapy with manual therapy in improving the range of motion, decreasing pain, and improving activity of daily living in patients with neck pain.

**Design:**

Double-blinded, randomized, experimental study.

**Patients:**

Women aged 45–65 with cervical pain due to osteoarthritis of the vertebral body and intervertebral disc.

**Methods:**

A total of 93 randomly selected females were included in the study. They were randomly divided into 2 groups. One received proprioceptive neuromuscular facilitation treatment and the other received manual therapy. To evaluate functional capabilities, the Oswestry Disability Index and range of motion measure were used. To evaluate changes in subjective experience of pain the Visual Analogue Scale was used.

**Results:**

In terms of the activities of daily living, pain, and range of motion of flexion, extension, lateral flexion to the right and left, and rotation to the right and left improvement in group I compared with group II was statistically significant (*p* < 0.05) at 2 weeks and 3 months’ follow-up.

**Conclusion:**

Treatment according to proprioceptive neuromuscular facilitation is a better method in comparison with manual therapy regarding improvement of pain, range of motion, and daily functioning in patients with cervical pain.

## LAY ABSTRACT

This study aimed to check whether a physiotherapy intervention called proprioceptive neuromuscular facilitation works better than manual therapy for women aged 45–65 with neck pain. A total of 93 women were randomly selected and split into 2 groups. One group received proprioceptive neuromuscular facilitation therapy, and the other had manual therapy. Dedicated tests were used to see how well each therapy improved the women’s range of motion in daily functions and to measure changes in pain. Before treatment, both groups had similar disability levels. After the treatment, it was found that the group with proprioceptive neuromuscular facilitation therapy showed better improvement in daily activities. In terms of pain, the same group also had better results. In conclusion, the study suggests that proprioceptive neuromuscular facilitation therapy is a more effective method than manual therapy for reducing pain, improving range of motion, and enhancing daily functioning in women with neck pain.

The annual incidence of neck pain in industrialized countries varies from 27% to 48%, which generates high costs and has become a key issue for the healthcare system and labour market (1, 2). Ylinen (3) reported that the prevalence of chronic neck pain is 7% to 22% in women and 5% to 16% in men. In particular, office workers run the risk of musculoskeletal system overload due to prolonged sitting positions with office equipment not suited to their needs (4). Cervical spine pain is common, expensive, and it impairs function, while the effectiveness of exercises as a physiotherapeutic intervention remains unclear (5). Following the increasing incidence of chronic neck pain, it is important to describe cost-effective, time-efficient, and patient-appropriate physiotherapeutic interventions (6–8).

A large variety of physiotherapeutic interventions is used for treating chronic neck pain: manual therapy (MAN.T), relaxation techniques, active exercises, transcutaneous electrical nerve stimulation (TENS), education, medications, and others (9, 10). International evidence-based clinical practice guidelines, which advocate a multimodal approach applying cervical manipulation or mobilization, neuromuscular exercise, stretching, strengthening, endurance training, aerobic conditioning, and patient education to improve function and reduce pain, are further supporting management of neck pain (11–13).

MAN.T is defined as a specialized area of physio-therapy for the management of neuro-musculoskeletal conditions, based on clinical reasoning, using highly specific treatment approaches including hands-on techniques and therapeutic exercises (14). There are several different manual therapy frameworks that are widely used with different approaches and treatment methods. Exelby (15) reports that MAN.T with joints mobilization was quite effective in the improvement of functional movements and decreasing pain. Farooq et al. (16) confirmed that cervical mobilization has a positive effect on pain, disability, endurance of neck muscles, and range of motion (ROM) in patients with chronic neck pain.

Proprioceptive training is popularly applied in rehabilitation settings for chronic neck pain patients but its effect on pain and function has been only poorly evaluated (17). A combination of strengthening, stretching, and aerobic exercises has the most beneficial effect on isometric strength, pain relief, and disability improvement with a general improvement in perceived well-being (18).

Among review articles concerning a wide range of current physiotherapeutic interventions, none of them included proprioceptive neuromuscular facilitation (PNF) in treating chronic neck pain. However, there is evidence for the effectiveness of PNF treatment for musculoskeletal disorders that contribute to neck pain (19).

The PNF concept uses movement patterns to restore lost function, where the patient is an active participant in the therapy. MAN.T focuses on improving functioning primarily by mobilizing the joints to restore mobility in the treated areas. In this method, the patient is more passive. The goal of this study was to compare the effects of PNF with MAN.T in improving ROM, decreasing pain, and improving activity of daily living (ADL) in patients with cervical pain.

## METHODS

### Setting

The parallel-group, single-centre, double-blinded randomized experimental study was conducted in Kraków Rehabilitation and Orthopaedic Centre’s outpatient clinic located at Aleja Modrzewiowa 22 in Kraków, Poland. This medical facility is ISO certified and accredited by the Minister of Health, which confirms compliance with quality standards for hospital treatment. The facility has extensive experience treating patients suffering from neck pain. The Bioethics Commission of Regional Medical Ethics of Physicians in Kraków, Poland expressed a positive opinion on conducting the studies (No. 71/KBL/OIL/2011). The study adheres to the Declaration of Helsinki for ethical principles for medical research involving humans. The study was registered at clinicaltrials.gov with a clinicaltrials.gov ID: NCT03683602.

### Study population

Patients admitted to the rehabilitation setting were screened and selected by a physical and rehabilitation medicine physician and on the basis of entry criteria they were either qualified or excluded. The following entry criteria for the patients were used: age 45–65, female gender, cervical pain, osteoarthritis of the vertebral body and intervertebral disc confirmed by X-ray, chronic pain lasting more than 13 weeks, preserved verbal contact, intact cognitive functions, and voluntary consent for the study. Patients were excluded if they had: birth or acquired deficits, cervical spine injury, osteoporosis, cervical spine instability, myelopathy, signs of nerve root compression of C1–C8, such as paresis, muscle loss, hyporeflexia, use of analgesics, anti-inflammatory drugs, or myorelaxants. All the patients involved provided written informed consent for participation in the study and conveyed baseline data: age, height, weight, and type of work performed (Table I).

**Table I:** Baseline characteristics

Group Item	I (PNF) n = 40	II (MAN.T) n = 40
Age, years, mean (SD)	56.3 (5.25)	55.75 (6.44)
Body mass index (kg/m^2^), mean (SD)	24.40 (3.04)	24.9 (3.17)
Type of work		
Pensioner, *n* (%)	3 (7.5)	5 (12.5)
Intellectual work, *n* (%)	33 (82.5)	28 (70)
Physical work, *n* (%)	4 (10.0)	7 (17.5)

SD: standard deviation; PNF: proprioceptive neuromuscular facilitation; MAN.T: manual therapy.

### Design

Prior to the first treatment patients who qualified for the study visited a single investigator who ran a randomization procedure and they were then randomly assigned to the PNF group or MAN.T group using simple randomization without stratification. Allocation of each participant was concealed until assignment. Specifying the group was done by drawing a sealed, opaque envelope: an envelope with an even number meant PNF treatment, while an odd number meant MAN.T. Four therapists treated patients, 2 with PNF and 2 with MAN.T. After randomization patients in both groups were blinded with regard to the kind of treatment received and the study hypothesis. They received information on who would be their physiotherapist.

Physiotherapists were assigned to a treatment group according to their expertise. Patients were treated by a therapist whom they drew, depending on the even or odd number. In group I the first therapist treated 22 patients, the second 18, among the 40 patients who completed the intervention. Therapists in group I completed PNF training and were experts in PNF therapy; they believed that this method is effective in treating cervical pain. In group II the first therapist treated 21 patients, the second 19. Therapists in group II completed MAN.T training according to the Kaltenborn–Evjenth framework; they were experts in MAN.T and they believed that this method was effective in treating cervical pain. Therapists taking part in the study were rehabilitation centre employees and all met the following requirements: holding a master’s degree in physiotherapy, 5–7 years’ professional experience as a physiotherapist working with adult patients with neck pain, completed training in PNF or MAN.T. Before the treatment, therapists received a protocol with specific guidelines and they were practically trained to deliver the intervention uniformly. Each therapist agreed to adhere to the steps of the physiotherapeutic protocol. One independent unannounced clinician assessed care providers if they followed the protocol.

The sample size was 80 patients and this decision was made based on a randomized controlled trial (20) where outcome measurements were collected at baseline, 2 and 4 weeks using the Numeric Pain Rating Scale (NPRS), the Patient-Specific Functional Scale (PSFS), and the Neck Disability Index (NDI) to evaluate the effectiveness of treatment of the cervical spine and subjective pain evaluation. For randomization 93 patients were included, assuming that about 10% would not finish the intervention for some reason. We tried to include as many patients as possible, considering the limitations of difficulties in selecting a larger group of willing people who would meet the inclusion criteria.

The effect size was calculated using Cohen’s d to show the practical significance of the obtained research results. Effect sizes were calculated for those comparisons that showed the statistical significance of differences.

### Data collection

A data collector responsible for measuring outcomes at baseline (T0), at 2 weeks (T1), and 3 months’ follow-up (T2) in the rehabilitation setting was blinded to the treatment group allocation. The data collector was not involved in the trial. The observer collecting data could not enter the exercise room while patients were treated and could not be present during randomization. Contact between a person responsible for the randomization process and the data collector, as well as between caregivers and the data collector, was avoided; this was monitored by the Head of the Physiotherapy Unit.

### Intervention

A neck pain-specific rehabilitation programme was implemented for all patients. PNF treatment was given to one group, and MAN.T treatment was given to the other. The patients were treated individually. Physical modalities such as TENS and laser therapy on the cervical spine were also administered to both groups. Treatment lasted for 2 weeks, with 10 x 45-minute rehabilitation sessions, 1 per day.

PNF is a rehabilitation concept widely used by physiotherapists, in which stimulation of the central nervous system is used to achieve the highest possible functional level. PNF techniques are used to help with the execution of functional movement by facilitation, inhibition, strengthening, or relaxing certain muscle groups by concentric, eccentric, and static muscle work (19). The PNF concept is based on 3 main pillars: philosophy, basic principles and procedures, as well as techniques. Philosophy is a guideline for a patient`s treatment. Techniques are used to help with the execution of functional movement by facilitation, inhibition, strengthening, or relaxing certain muscle groups by concentric, eccentric, and static muscle work. Basic principles enable optimal, goal-oriented, and comprehensive stimulation of the central nervous system directed at restoring or improving activities of daily living (21).

In this study, joint mobilization according to Kaltenborn–Evjenth, one of the MAN.T frameworks, was used. The Kaltenborn–Evjenth method consists of several elements. The first is functional assessment using manual tests to determine the source of pain. The next elements are therapy methods tailored to the patient’s needs, such as: soft tissue mobilization techniques, joint mobilization techniques, neural tissue mobilization techniques, and exercises (22).

The joints were mobilized with low-velocity passive movements in the whole or end range. Spinal manipulation was not used.

Details of the interventions provided for both groups are presented in Table II. The only component these 2 treatment approaches had in common was re-education of postural control.

**Table II:** Details of the interventions for both groups

Treatment in group I (PNF) included the following techniques used in PNF:	Treatment in group II (MAN.T) included the following techniques used in MAN.T:
- neck movement patterns: neck flexion–lateral flexion–rotation, neck extension–lateral flexion–rotation with the technique Combination of Isotonic, starting position: supine, duration 6 min	- mobilization of the cervicothoracic junction, in a sitting position, duration 5 min
- upper extremity movement patterns: flexion–abduction–external rotation and extension–adduction–internal rotation with the technique Combination of Isotonic, starting position: lying on back, duration 6 min	- cervical segmental mobilization (flexion, extension, coupling movement), supine, duration 5 min
- neck patterns: neck flexion–lateral flexion–rotation, neck extension–lateral flexion–rotation with the technique Hold-Relax, starting position: sitting, duration 6 min	- isometric exercises of cervical spine, supine, duration 5 min
- scapula movement patterns: anterior elevation, posterior depression with techniques Stabilizing Reversals and Contract-Relax, duration 6 min	- post-isometric muscle relaxation for the levator scapulae and trapezius muscles, duration 5 min
- re-education of postural control – performing head retraction movements while sitting in front of a mirror, duration 6 min	- traction of the cervical spine, supine, duration 4 min
	- re-education for postural control, performing head retraction movements while sitting in front of a mirror, duration 6 min

PNF: proprioceptive neuromuscular facilitation; MAN.T: manual therapy.

**Fig. 1:**
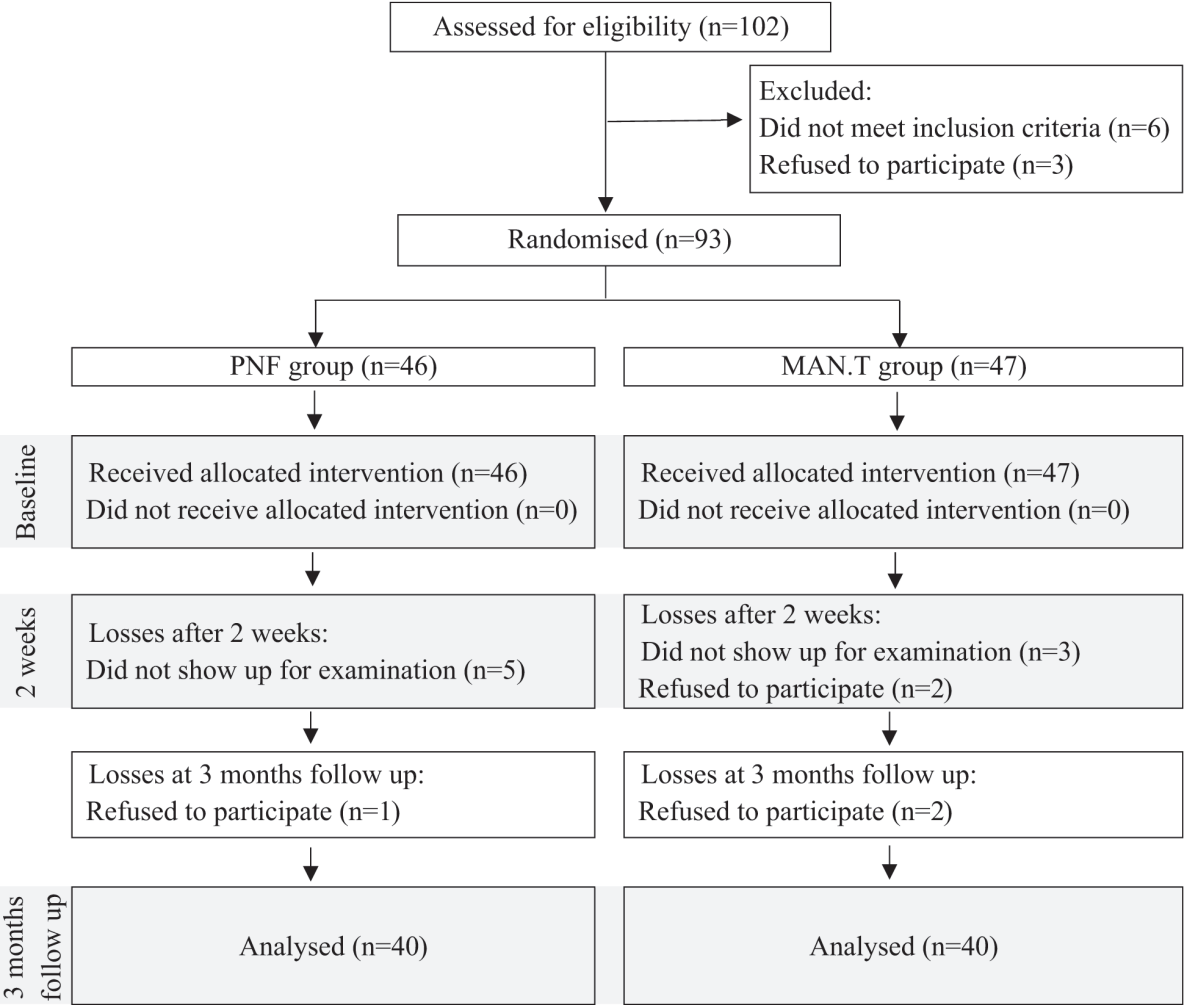
Flowchart of study participation. PNF: proprioceptive neuromuscular facilitation; MAN.T: manual therapy.

Fig. 1 summarizes patients who were evaluated, recruited, randomized, and observed. Baseline characteristics of the patients are given in Table I. Most patients in both groups were professionally active and most of them were office workers. At baseline, groups were comparable concerning age, BMI (*p* > 0.05) and type of work performed (Table I).

### Outcomes

Data were collected concerning age, body mass index (BMI), and type of work. Improvement in therapy was measured with standardized scales. Measurements were taken at T0, T1, and T2. To observe the primary outcome of ADL the Oswestry Disability Index (ODI) was used; the secondary outcomes of ROM and subjective experience of pain were monitored with a Gulick tape measurement in centimetres and the Visual Analogue Scale (0–10) (VAS) respectively.

To evaluate the effects of the 2 applied approaches, measurement outcomes that have been shown to be reliable have been utilized. ODI can be used for the evaluation of both the cervical and lumbar spine. ODI consists of 10 sections, 8 related to ADL and 2 related to pain. The sections are presented in Table III. Each question has 6 possible answers, rated from 0 to 5 points: 0 means that the patient has no problems performing a given activity, while 5 means the greatest problem in a given activity (23). It is the most common scale used for disability evaluation of patients with neck pain (24, 25) with validation and cross-cultural adaptation of the Polish version with excellent test–retest reliability, an intraclass correlation coefficient at 0.97, and standard error of measurement at 3.54 (26).

**Table III:** Median and quartile of pain, Oswestry Scale, ROM of subjects in the PNF and MAN.T group at baseline at 2 weeks and 3 months’ follow-up with differences between the groups

Group	I (PNF)				II (MAN.T)				Differences between the groups		
	Baseline Median (IQR)	2 weeks Median (IQR)	3 months follow up Median (IQR)	*p*-value	Baseline Median (IQR)	2 weeks Median (IQR)	3 months follow up Median (IQR)	*p*-value	*p*-value*	*p*-value**	*p*-value***
Oswestry Scale for neck											
Pain intensity	2 (1–2)	1 (1–1)	1 (1–1)	0.0001^c^	2 (1–2)	2 (1–2)	2 (1–2)	0.0388^b^	0.319^b^	**0.0001**^b^ (1.05)	**0.0001**^b^ (0.87)
Personal Care	1 (0–1)	0 (0–1)	1 (0–1)	0.0007^c^	1 (0–1.5)	1 (0–1)	1 (0–1.5)	0.1561^b^	0.118^b^	**0.002**^b^ (0.73)	0.082^b^
Lifting	1 (1–2)	1 (0–1)	1 (0–1)	0.0001^c^	2 (1–3)	1 (1–2)	2 (1–2)	0.0001^b^	**0.008**^b^ (0.32)	**0.0001**^b^ (1.06)	**0.0001**^b^ (0.96)
Reading	2 (1–3)	1 (1–1)	1 (1–1.5)	0.0001^c^	2 (1.5–3)	1 (1–2)	2 (1–2)	0.0001^b^	0.644^b^	**0.003**^b^ (0.70)	**0.002**^b^ (0.75)
Headaches	2 (2–3)	1 (1–1)	1 (1–1)	0.0001^c^	2 (1–3)	2 (1–3)	2 (1–3)	0.0001^b^	0.376^b^	**0.0001**^b^ (0.97)	**0.0001**^b^ (0.83)
Concentration	1 (0–1)	0 (0–1)	1 (0–1)	0.0001^c^	1 (1–2)	1 (0.5–1)	1 (1–1.5)	0.0082^b^	0.073^b^	**0.0015**^b^ (0.74)	**0.0226**^b^ (0.54)
Work	2 (1–3)	1 (0.5–1)	1 (0.75–2.0)	0.0001^c^	2 (1–2.5)	1 (1–2)	1 (1–2)	0.0001^b^	0.715^b^	0.099^b^	0.169^b^
Driving	2 (1–2)	1 (0–1)	1 (0.5–1)	0.0001^c^	2 (1–2)	1.5 (1–2)	1.5 (1–2)	0.0001^b^	0.667^b^	**0.0001**^b^ (1.04)	**0.0001**^b^ (0.96)
Sleeping	2 (1–2)	1 (0–1)	1 (1–1)	0.0001^c^	2 (1–3)	1 (0.5–2)	1 (1–2)	0.0001^b^	0.686^b^	0.0682^b^	**0.0395**^b^ (0.49)
Recreation	2 (1–2)	1 (0–1)	1 (0–1)	0.0001^c^	2 (1–2)	1 (1–2)	1 (1–2)	0.0001^b^	0.436^b^	**0.0061**^b^(0.60)	**0.0026**^b^ (0.68)
Total number of points	16 (13–17.5)	7 (6–9)	9 (7–11)		17 (12–22)	13 (10–16)	14 (11–17)		0.111^a^	**0.0001**^a^ (1.12)	**0.0001**^a^ (1.01)
Range of motion											
Flexion	2 (1.25–2.5)	3.5 (2.5–4)	3 (2.5–4)	0.0001^c^	2 (2–3)	3 (2.5–3.25)	3 (2–3)	0.0001^b^	**0.048**^b^ (0.34)	0.0833^b^	0.294^b^
Extension	4 (3–5)	7 (5.5–7)	6 (5–7)	0.0001^c^	5 (4–6)	5 (4–7)	5 (4–6.25)	0.0001^b^	0.334^b^	**0.0155**^b^ (0.52)	**0.0355**^b^ (0.47)
Lateral flexion to the right	2 (1–2)	3 (2–3.5)	3 (2–3)	0.0001^c^	2 (1.25–3)	2.75 (2–3.5)	2 (1.75–3)	0.0001^b^	0.326^b^	0.262^b^	0.254^b^
Lateral flexion to the left	4 (3.5–5)	6 (5–7)	6 (5–7)	0.0001^c^	2 (1.25–3)	2.75 (1.75–3)	2 (1.5–3)	0.0001^b^	0.145^b^	0.096^b^	**0.047**^b^ (0.30)
Rotation to the left	4 (3.5–5)	6 (5–7)	6 (5–7)	0.0001^c^	4.25 (4–6)	5 (4–6)	5 (4–6)	0.0001^b^	0.613^b^	**0.013**^b^ (0.56)	**0.0159**^b^ (0.55)
Rotation to the right	4 (3–5)	6 (4–7)	5.75 (4–7)	0.0001^c^	5 (4–6)	5 (4–7)	5 (4–6)	0.0001^b^	0.135^a^	0.355^a^	0.160^a^
VAS											
Pain intensity	3 (2.5–4.75)	1 (1–2)	2 (1–2)	0.0001^c^	4 (3–5)	3 (2–4)	3 (2–4)	0.0001^b^	0.101^a^	**0.0001**^b^ (1.27)	**0.0001**^b^ (1.12)

ROM: range of motion; PNF: proprioceptive neuromuscular facilitation; MAN.T: manual therapy; VAS: Visual Analogue Scale; IQR: interquartile range.

Significant difference between the two groups (*p* < 0.05).

a Student’s *t*-test.

b Mann–Whitney *U* test.

c Friedman ANOVA test.

* Effect size, group I vs group II baseline.

** Effect size, group I vs group II at 2 weeks.

*** Effect size, group I vs group II at 3 months’ follow-up.

ROM of the cervical spine was evaluated with a measuring tape. The measurement was made with a Gulick tape in a sitting position. The reliability of this method has been demonstrated by Asha and Pryor (27).

One of the most commonly used and valid scales for pain evaluation is VAS (28, 29). It is easy to use, it does not require any verbal or reading abilities, and it is comprehensive enough (30). A patient is asked to indicate his/her perceived pain intensity (most commonly) along a 100 mm horizontal line, and this rating is then measured from the left side (31). VAS has proved to have a good intra-tester reliability with an ICC of > 0.75 (32).

### Statistical analysis

Descriptive statistics were calculated: mean, standard deviation, median, Q25, Q75, minimum, and maximum. In order to check the conformity of the distribution of the analysed variables to a normal distribution, the Shapiro–Wilk test was applied. The analysis of differences between the 2 groups for quantitative variables with a normal distribution was performed using Student’s *t*-test; for quantitative variables with a distribution other than normal, or ordinal, the Mann–Whitney *U* test was used. For comparisons of qualitative variables between the two groups, the χ^2^ test was used. To test whether there were changes over time in the variables analysed (T0 vs T1 vs T2), the Friedman ANOVA test was used. To test whether there were differences between groups for changes (T2 minus T0, T1 minus T0), the Mann–Whitney *U* test was used.

The analysis was performed using STATISTICA 13 PL (https://www.statsoft.de/en/data-science-applications/tibco-statistica/), with *p* < 0.05 as the level of statistical significance (33).

## RESULTS

Differences between the 2 groups in ODI, ROM of the cervical spine, and VAS outcomes between T0, T1, and T2 are shown in Table III and Appendix S1.

**Fig. 2:**
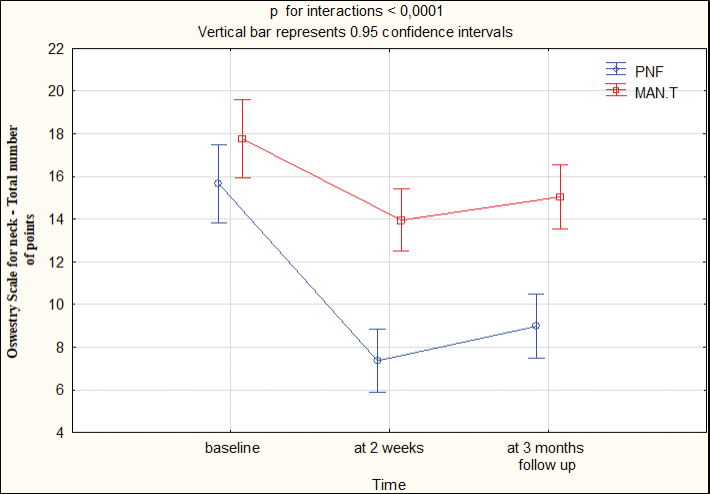
Oswestry Disability Index for neck: total numbers at baseline, 2 weeks, 3 months’ follow-up. PNF: proprioceptive neuromuscular facilitation; MAN.T: manual therapy.

Group I and group II were comparable at T0 for all outcome measures (ODI, ROM, and VAS); all outcomes at T0 were calculated with *p* > 0.05. ODI improved in group I by 8 points and in group II by 4 points at T1 and T2. The differences between the groups were statistically significant (*p* < 0.05) in favour of the group I at T1 and T2 (Table III, Appendix S1). Fig. 2 presents the outcomes.

**Fig. 3:**
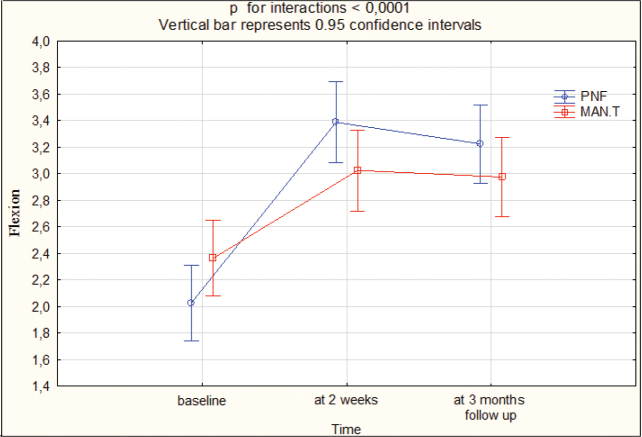
Range of motion: flexion at baseline, 2 weeks, 3 months’ follow-up. PNF: proprioceptive neuromuscular facilitation; MAN.T: manual therapy.

**Fig. 4:**
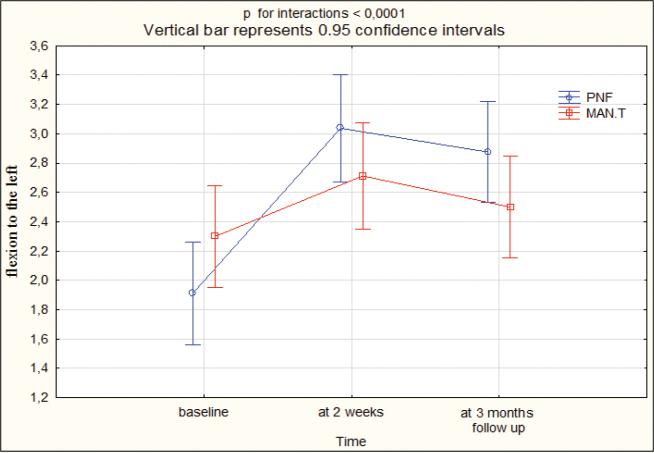
Range of motion: flexion to the left at baseline, 2 weeks, 3 months’ follow-up. PNF: proprioceptive neuromuscular facilitation; MAN.T: manual therapy.

**Fig. 5:**
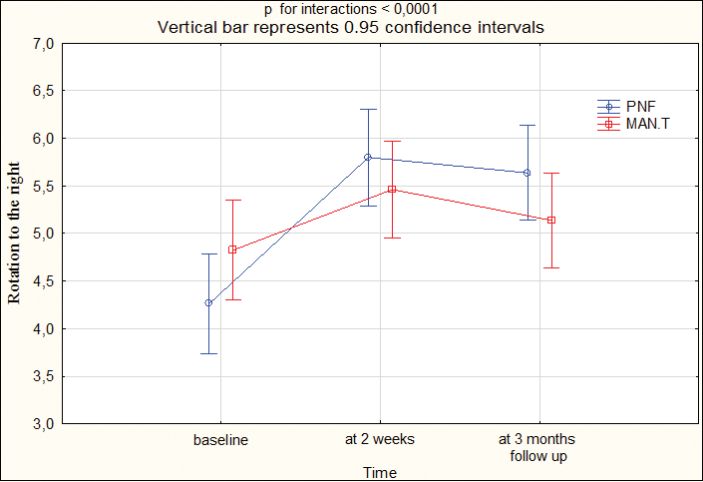
Range of motion: rotation to the right at baseline, 2 weeks, 3 months’ follow-up. PNF: proprioceptive neuromuscular facilitation; MAN.T: manual therapy.

At T1, the cervical spine ROM improved by 1.5 cm for flexion, 2.0 cm for extension, 1 cm for lateral flexion to the right, 1 cm for lateral flexion to the left, 2 cm for rotation to the left, 2 cm for rotation to the right in group I, while in group II this was 1 cm for flexion, 1 cm for extension, 0.5 cm for lateral flexion to the right, 0.5 cm for lateral flexion to the left, 1 cm for rotation to the left, 1 cm for rotation to the right. At T2, ROM improved by 1 cm for flexion, 2 cm for extension, 1 cm for lateral flexion to the right, 1 cm for lateral flexion to the left, 1.25 cm for rotation to the left, 1.5 cm for rotation to the right in group I, while in group II this was 1 cm for flexion, 0.5 cm for extension, 0.25 cm for lateral flexion to the right, 0 cm for lateral flexion to the left, 0.5 cm for rotation to the left, 0.5 cm for rotation to the right. The differences between the groups were statistically significant (*p* < 0.05) in favour of group I at T1 and T2 (Table III, Appendix SI). Figs 3–5 present the outcomes.

**Fig. 6:**
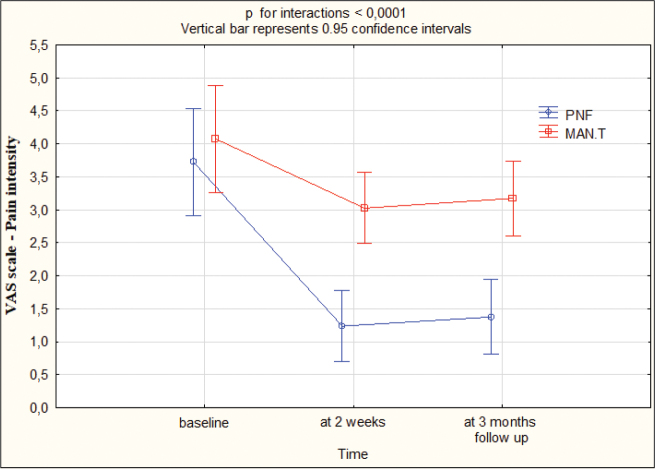
VAS: pain intensity at baseline, 2 weeks, 3 months’ follow-up. PNF: proprioceptive neuromuscular facilitation; MAN.T: manual therapy; VAS: Visual Analogue Scale.

VAS improved by 2 cm in group I and by 1 cm in group II at both T1 and T2. The differences between the groups were statistically significant (*p* < 0.05) in favour of group I at T1 and T2 (Appendix S1, Table III). Fig. 6 presents the outcomes.

Although no significant side effects were noted in this investigation, patients in both groups occasionally reported experiencing greater pain during treatment.

## DISCUSSION

In this study, analysis of results showed that PNF was more effective in this setting. The PNF group in comparison with the MAN.T group had a more favourable effect on pain (VAS), quality of ADL (ODI), and ROM of the cervical spine in patients with neck pain after 2 weeks’ treatment as well as 3 months after finishing the therapy. The effect size confirms the hypothesis for ODI and VAS, and to a lesser extent for ROM. For ODI and VAS the effect sizes are high while for ROM the value is medium.

Strengthening of deep neck flexors was implemented in the PNF group, addressed with neck movement patterns: neck flexion–lateral flexion–rotation, neck extension–lateral flexion–rotation with the Combination of Isotonic technique. On the basis of the results obtained in the study, it can be concluded that group I, where these exercises were included, was better in comparison with group II, where isometric exercises of the cervical spine were included. Functional disturbances of deep neck flexors occur in patients who suffer from pain in this part of the spine (34). According to Falla et al. (35) there is a relationship between the endurance of these muscles and intensity of pain. The authors conclude that the weaker the deep flexors the more severe the neck pain. Boyd-Clark et al. (36) emphasize the role of deep neck flexors in keeping the proper position and stability of the cervical spine. They also indicate that deep neck flexors together with muscles of the shoulder girdle play an important role in neck stabilization and keeping the weight of the head against gravity. The use of special strengthening exercises as a routine practice in the case of chronic neck pain can be beneficial (37). Patients report less neck pain after strengthening exercises as a result of stronger neck flexors and extensors (38). Lluch et al. (39) showed improvement in pain and disability in patients training deep neck flexors. However, it should be stressed that the treatment lasted 6 weeks, 4 weeks longer than in our research. In the study of Graaf and Schmitt (40) patients reacted positively to the training of deep neck flexors; they improved ROM of the cervical spine, vertigo, pain, and limitations of ADL. However, Cho et al. (41) stated in their studies that a combination of upper cervical and upper thoracic spine mobilization indicated better overall short-term outcomes in decreasing pain, respiratory function, and the global rating of change compared with deep cervical flexors exercise in individuals with forward head posture. Moghadam et al. (42) presented results where they showed no significant difference between the performance of the deep neck flexors during the craniocervical flexion test in forward head posture and normal head posture individuals, which undermines a common view of weak deep neck flexors in people with forward head posture.

There is scientific research available that progressive shoulder–neck exercise might provide a positive effect on deep and superficial neck muscle strength in patients with chronic neck pain (43). In the PNF group, exercises including PNF movement patterns of the neck and shoulder girdle were implemented, which could have had an impact on the results in group I.

Considering neck pain problems through the prism of deficits of stabilization/dysfunction of deep neck and shoulder girdle stabilizers, PNF can undoubtedly bring more benefits. It requires a greater engagement of the patient in the treatment and the effect is also better position of the head, neck, and shoulder girdle.

MAN.T is popular among therapists and patients. Several reports confirm the benefits of using it (16, 44). It is expected that these benefits were registered in patients without advanced degenerative changes. In follow-up observations these effects lasted for a shorter time because of lack of influence on head position patterns (45). Mobilizing intervertebral joints might have a physiological effect, influencing joint lubrication and nutrition for the cartilage. Furthermore, joint mobilization might influence the neurophysiological afference from joint mechanic receptors, altering pain experience (46).

In order to increase mobility in the PNF group the technique Hold-Relax was used for the neck and Contract-Relax for the shoulder girdle. Hold-Relax is resisted isometric contraction of the shortened muscle followed by relaxation; Contract-Relax is resisted isotonic contraction of the restricting muscles followed by relaxation and movement into the new increased range. Analysis of our research showed that in the group treated with PNF, there was a greater improvement in ROM in comparison with the MAN.T group. Hold-Relax and Contract-Relax are stretching techniques in the PNF concept used for muscle elasticity improvement, and it was shown that they have a positive influence on passive and active ROM. PNF increases ROM by improving muscle length and neuro-muscular efficacy. It was found that stretching with PNF improves ROM in both trained and untrained individuals (47). There is a lack of evidence indicating the efficacy of the PNF technique Hold-Relax on the improvement of neck mobility. However, there are many studies showing the effectiveness of the stretching technique Hold-Relax on a glenohumeral internal-rotation deficit, quadriceps flexibility, hamstring flexibility, hip flexors, and in patients with post-traumatic elbow stiffness (48–51). In the MAN.T group, the post-isometric muscle relaxation technique was used, which is a form of muscle energy technique in which the patient’s muscles are moved in a particular direction against the counterforce of the therapist, which is mediated by the Golgi tendon organ when the muscle contracts isometrically. Khan et al. (52) demonstrated that patients with non-specific neck pain can benefit from post-isometric relaxation with significant improvement in pain, ADL, cervical ROM, and quality of life compared with myofascial release therapy.

Hutting et al. (53) claim that manipulation and mobilization of the cervical spine are good interventions in treating patients with headaches and/or neck pain. However, benefits are accompanied by a potential but rare risk of serious adverse events including damage to brain blood vessels. Therefore, the authors attempted to compare the PNF method with MAN.T to find an equally effective treatment with fewer side effects and/or risks. PNF is a popular method in Poland; it is part of the curriculum in academic programmes, is funded by the National Health Fund, and is often prescribed by physical and rehabilitation medicine physicians. It was impossible to have a non-intervention control group because patients with rehabilitation referrals had to receive therapy as the hospital cannot refuse treatment to a patient with an appropriate referral.

### Limitations of the study

A limitation of this study is that only women were involved and patients over 65 years old were not included, knowing that degenerative changes in the cervical segments are more severe with age. For the evaluation of ROM, a measuring Gulick tape was used and not an inclinometer, which is a more effective tool (54). Maintaining the results 3 months after treatment could be associated with the patient’s lifestyle rather than with the therapy used. The therapeutic effect could be a reason for the development of good relations between participants and care providers, not with the therapy used. Another limitation was that 13 patients who were randomized did not complete the intervention and they were not evaluated on the day they resigned. An ITT “intention-to-treat” analysis was not done, which reduces the credibility of the results obtained. The rehabilitation procedure was consistent as far as possible with the protocol regarding the kind of exercises, while the number of repetitions was suited to the patients’ needs, which may interfere with the results.

The study was performed in a hospital where on average 200 patients are treated every 2 weeks. Due to this high volume, restrictive inclusion criteria could be used. In connection with such a large number of patients, the care providers had great experience of treating patients with neck pain. It would not be easy to repeat this study in a smaller centre and therapists with such high qualifications, thus the intervention evaluated should be reserved for high-volume centres. The duration of the therapy, which was performed on 10 days in 2 weeks, every day from Monday to Friday, was specific to the rehabilitation centre in which the study was done. It is not known if this form of therapy where the patient is being treated every day is more effective than 10 sessions over a longer time period with a frequency of 1, 2, or 3 times a week. It seems appropriate to conduct research where patients will be treated for a longer time and effects will be evaluated in the long term.

### Conclusion

The clinical implication from these observations is that during the treatment planning for women with neck pain caused by osteoarthritis the following should be considered: strengthening exercises of deep neck flexors, stretching exercises of neck and shoulder girdle muscles, performing neck and shoulder movement patterns, exercise for the shoulder joint, and re-education of postural control according to the PNF concept. The implication for understanding the nature and cause of chronic neck pain is that loss of postural control due to weakening of the deep neck flexors leads to the overloading of facet joints in the cervical spine (55).

In addition, further analysis of PNF treatment would be recommended, for example, to compare which therapy is more beneficial for patients with cervical pain: PNF connected with MAN.T or treatment with PNF only. Treatment according to PNF principles is a better method in comparison with MAN.T regarding improvement of pain, ROM, and functioning in daily living in patients with neck pain.

## Supplementary Material

PROPRIOCEPTIVE NEUROMUSCULAR FACILITATION THERAPY VERSUS MANUAL THERAPY IN PATIENTS WITH NECK PAIN: A RANDOMIZED CONTROLLED TRIAL

## Trial details

The study was registered in clinicaltrials.gov with a clinicaltrials.gov ID: NCT03683602.
