# Supplementary material for: PROPRIOCEPTIVE NEUROMUSCULAR FACILITATION THERAPY VERSUS MANUAL THERAPY IN PATIENTS WITH NECK PAIN: A RANDOMIZED CONTROLLED TRIAL
Source: J Rehabil Med. 2024 Sep 5;56:40002. doi: 10.2340/jrm.v56.40002 (PMC11558861; doi:10.2340/jrm.v56.40002)
Supplement: Supplementary file 1 — PROPRIOCEPTIVE NEUROMUSCULAR FACILITATION THERAPY VERSUS MANUAL THERAPY IN PATIENTS WITH NECK PAIN: A RANDOMIZED CONTROLLED TRIAL [file JRM-56-40002-s1.pdf]

Supplementary material has been published as submitted. It has not been copyedited, or typeset by Journal of Rehabilitation Medicine

Appendix S1. Differences in the examined parameters between the groups in the time period 3 months follow up vs baseline and 2 weeks vs baseline

	Group I (PNF) Me (Q1-Q3)	Group II (MAN.T) Me (Q1-Q3)	p-value (effect size)
**ODI for neck**			
Pain intensity 3 months follow up vs baseline	-1(-1-0)	0(0-0)	**0.0005[a]** (0.81)
Pain intensity 2 weeks vs baseline	-1(-1-0)	0(0-0)	**0.0001[a]** (1.30)
Personal Care 3 months follow up vs baseline	0(0-0)	0(0-0)	0.8814[a]
Personal Care 2 weeks vs baseline	0(-1-0)	0(0-0)	0.1248[a]
Lifting 3 months follow up vs baseline	-0.25(-1-0)	0(-1-0)	0.5129[a]
Lifting 2 weeks vs baseline	-1(-1-0)	-0.5(-1-0)	0.3998[a]
Reading 3 months follow up vs baseline	-1(-1-0)	0(-1-0)	**0.0077[a]** (0.63)
Reading 2 weeks vs baseline	-1(-1.5,-1)	-1(-1-0)	**0.0147[a]** (0.59)
Headaches 3 months follow up vs baseline	-1(-1.5,-1)	0(-1-0)	**0.0015[a]** (0.67)
Headaches 2 weeks vs baseline	-1(-2,-1)	-0.5(-1-0)	0.0175[a]
Concentration 3 months follow up vs baseline	0(-1-0)	0(0-0)	0.4500[a]
Concentration 2 weeks vs baseline	0(-1-0)	0(0-0)	0.0841[a]
Work 3 months follow up vs baseline	-1(-1.25-0)	0(-1-0)	**0.0161[a]** (0.54)
Work 2 weeks vs baseline	-1(-2-0)	0(-1-0)	**0.0189[a]** (0.56)
Driving 3 months follow up vs baseline	-1(-1,-1)	0(-1-0)	**0.0001[a]** (0.91)
Driving 2 weeks vs baseline	-1(-1,-1)	0(-1-0)	**0.0001[a]** (1.10)
Sleeping 3 months follow up vs baseline	-1(-1-0)	0(0-0)	**0.0003[a]** (0.78)
Sleeping 2 weeks vs baseline	-1(-1,-1)	0(-1-0)	**0.0003[a]** (0.83)
Recreation 3 months follow up vs baseline	-1(-1-0)	0(-1-0)	**0.0033[a]** (0.78)
Recreation 2 weeks vs baseline	-1(-1-0)	0(-1-0)	**0.0101[a]** (0.64)
Total number of points 3 months follow up vs baseline	-7(-9,-4.5)	-3(-4,-1)	**0.0001[a]** (1.27)
Total number of points 2 weeks	-8(-9.75,-7)	-4(-5,-2)	**0.0001[a]** (1.73)

vs baseline			
**ROM**			
Flexion 3 months follow up vs baseline	1(1-1.5)	1(0-1)	**0.0003**[a] (0.94)
Flexion 2 weeks vs baseline	1.5(1-2)	1(0.25-1)	**0.0001**[a] (1.15)
Extension 3 months follow up vs baseline	2(1-2)	0.5(0-1)	**0.0001**[a] (1.29)
Extension 2 weeks vs baseline	2(1.75-3)	1(0-1)	**0.0001**[a] (1.60)
Lateral flexion to the right 3 months follow up vs baseline	1(0.5-1)	0.25(0-1)	**0.0010**[a] (0.64)
Lateral flexion to the right 2 weeks vs baseline	1(1-1)	0.5(0-1)	**0.0004**[a] (0.86)
Lateral flexion to the left 3 months follow up vs baseline	1(1-1)	0(0-0.5)	**0.0001**[a] (1.31)
Lateral flexion to the left 2 weeks vs baseline	1(1-1)	0.5(0-0.5)	**0.0001**[a] (1.64)
Rotation to the left 3 months follow up vs baseline	1.25(0.75-2)	0.5(0-1)	**0.0002**[a] (0.88)
Rotation to the left 2 weeks vs baseline	2(1-2)	1(0-1)	**0.0001**[a] (1.11)
Rotation to the right 3 months follow up vs baseline	1.5(1-2)	0.5(0-0.5)	**0.0001**[a] (1.20)
Rotation to the right 2 weeks vs baseline	2(1-2)	1(0-1)	**0.0001**[a] (1.07)
**VAS**			
Pain intensity 3 months follow up vs baseline	-2(-2.25,-1)	-1(-1,-0.5)	**0.0001**[a] (0.83)
Pain intensity 2 weeks vs baseline	-2(-2.75,-1.25)	-1(-1,-1)	**0.0001**[a] (0.78)

ODI: Oswestry Disability Index; ROM: range of motion; PNF: proprioceptive neuromuscular facilitation; MAN.T: manual therapy; VAS: Visual Analogue Scale; Q1: bottom quartile; Q3: upper quartile; Me: median
Significant difference between the two groups ($p < 0.05$). Statistically significant values are marked in bold.
[a]Mann-Whitney U test
